# A Hybrid Frequency Estimation Framework for Long-Range FMCW LiDAR Under Low-SNR Conditions

**DOI:** 10.3390/s26185959

**Published:** 2026-09-20

**Authors:** Yating Fang, Xiaohai Yu, Chaochao Zhang, Jia He, Yuanying Zhang, Hua Zheng, Xiaoqin Zhu

**Affiliations:** Fujian Provincial Key Laboratory for Photonics Technology, Fujian Provincial Engineering Technology Research Center of Photoelectric Sensing Application, College of Photonic and Electronic Engineering, Fujian Normal University, Fuzhou 350117, China

**Keywords:** FMCW LiDAR, low-SNR conditions, frequency estimation, OMP, GWO

## Abstract

Frequency-modulated continuous-wave (FMCW) light detection and ranging (LiDAR) has become increasingly significant for long-distance ranging fields. However, the beat signal is vulnerable to noise interference during detection, resulting in a low signal-to-noise ratio (SNR) and inaccurate ranging results. Although traditional methods have improved accuracy through various denoising and spectral analysis algorithms, they often struggle to maintain robust performance in long-range scenarios under low-SNR conditions. To address this challenge, this paper proposes the HOIGL, a hybrid frequency estimation algorithm which integrates Orthogonal Matching Pursuit (OMP) for initial value guidance and Gray Wolf Optimization (GWO) for local search. Specifically, by leveraging the frequency-domain sparsity of the signal, OMP performs piecewise sparse representation to yield a coarse initial frequency, within which GWO conducts a refined continuous local search to overcome dictionary discretization bias based on a custom fitness function. Monte Carlo simulations in MATLAB demonstrate that the proposed algorithm achieves an RMSE of 2.63 m compared to over 84.34 m for other methods at −20 dB SNR, while maintaining a low and stable processing time of around 0.498 s. Finally, fiber-optic-link experimental results verify the performance of HOIGL for hundred-meter-scale range detection.

## 1. Introduction

Light detection and ranging (LiDAR), as a high-precision active optical remote sensing technology, has the advantages of high measurement accuracy, high spatial resolution, and strong anti-interference ability [1]. It is widely applied in autonomous driving [2], robot navigation [3], geographic mapping [4] and other fields. As the mainstream coherent LiDAR technology, frequency-modulated continuous-wave (FMCW) LiDAR combines laser interference technology and FMCW technology, wherein the laser frequency varies linearly over time. Through optical interference, a beat frequency signal is generated, from which the measurement result is derived via frequency demodulation [5]. Compared to incoherent-detection LiDAR, FMCW LiDAR has the advantages of robustness against ambient light, low transmit power, extensive measurement range, and simultaneous ranging and velocity measurement, making it a growing research focus in recent years [6]. However, long-distance detection presents substantial challenges. Due to the weak intensity of echo signal, coupled with environmental and system-induced noise, the signal-to-noise ratio (SNR) of the beat frequency signal is relatively low [7]. This significantly increases the difficulty of subsequent signal processing and analysis, severely affecting the accuracy and completeness of spectral peak extraction, thereby compromising ranging precision [8]. Furthermore, the traditional Fast Fourier Transform (FFT) algorithm suffers from inherent limitations, such as spectral aliasing, staircase effect and truncation effect, which inevitably introduce errors in spectral analysis.

To improve the accuracy of frequency extraction, current research has proposed methods for noise suppression, including wavelet threshold denoising [9], Empirical Mode Decomposition (EMD) algorithm [10], and Variational Mode Decomposition (VMD) algorithm [11], along with their respective enhancements [12,13]. Additionally, spectrum refinement techniques such as zero-padding in the time domain [14], the Chirp-z transform (CZT) algorithm [15], the Zoom-FFT (ZFFT) algorithm [16], the Rife algorithm [17] and the Quinn algorithm [18] have been introduced to reduce ranging errors. Nevertheless, the aforementioned denoising algorithms fail to provide adequate performance for long-distance detection under low-SNR conditions. Moreover, spectrum refinement algorithms remain fundamentally constrained by the precision limitations of the underlying FFT. Furthermore, the conventional step-by-step signal processing approach of first applying denoising followed by FFT not only increases system complexity and reduces efficiency, but may also impact the final results due to error accumulation across stages.

To address this challenge, this paper proposes HOIGL, a hybrid frequency estimation algorithm which integrates Orthogonal Matching Pursuit (OMP) [19] for initial value guidance and Gray Wolf Optimization (GWO) [20] for local search. This algorithm enables robust frequency estimation even in the presence of strong noise, making it exceptionally well-suited for long-range FMCW LiDAR applications under low-SNR conditions. Specifically, leveraging the natural sparsity of the beat frequency signal in the frequency domain compared to noise, the OMP algorithm is utilized for piecewise sparse representation on the signal to obtain coarse frequency estimates for each segment. By taking the median of all frequencies, we obtain the rough estimated initial frequency. Then, to overcome the discretization bias inherent in the OMP dictionary, the GWO algorithm is introduced to perform a refined local continuous search within the neighborhood of the initial frequency. We further design a complex fitness function that integrates signal correlation, residual energy, and spectral peak intensity to optimize frequency selection. At the same time, we adopt strategies such as small population, few iterations, and dynamic boundary constraints to effectively balance high estimation accuracy with computational efficiency for the FMCW LiDAR system. Monte Carlo simulations in MATLAB and fiber-optic-link experimental results show that the measurement accuracy of the HOIGL algorithm is superior to that of other algorithms. These results demonstrate the potential of the proposed algorithm for long-distance ranging under low-SNR conditions in the future.

## 2. Theoretical Principle

### 2.1. Principle of FMCW LiDAR Ranging

**Figure 1:**
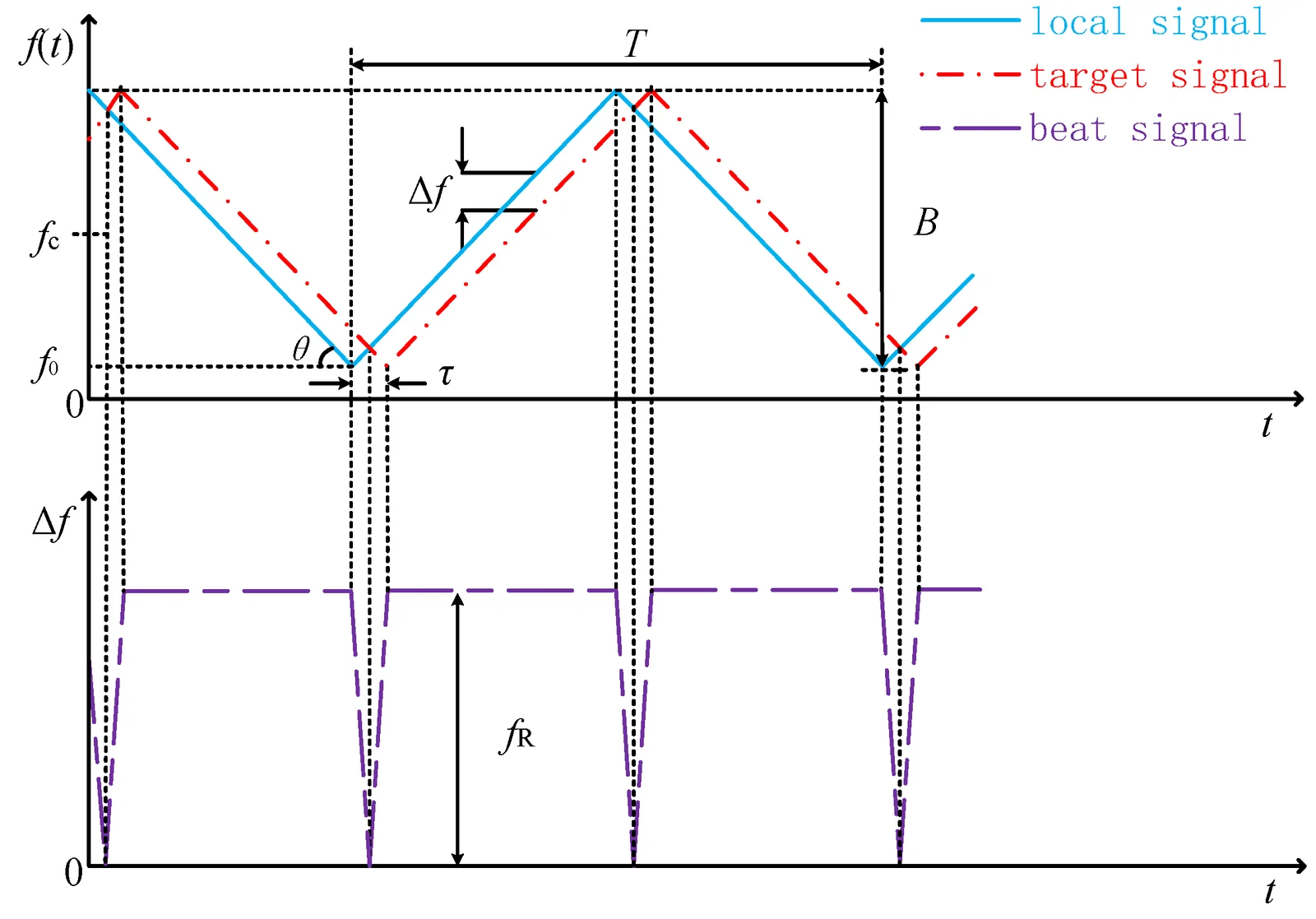
Principle diagram of FMCW LiDAR for detecting stationary targets.

Triangular wave modulation simplifies the demodulation of distance-induced and Doppler frequency shifts, facilitating simultaneous ranging and velocity measurement [6]. The laser in this study adopts triangular wave modulation. Under ideal conditions, the fundamental principle for measuring the distance to a stationary target is illustrated in Figure 1.

The signal frequency varies linearly and periodically over time, with equal rise and fall times. In the figure, *f*_c_ denotes the central frequency of the light source, *B* represents the modulation bandwidth, *T* represents the sweep period and  tan(θ) stands for the sweep rate.

In an FMCW LiDAR ranging system, the beat frequency electrical signal received by the photodetector can be simplified as

(1) $$U\left(t\right)=A_{0}\cos\left(2\pi f_{R}t+\varphi_{0}\right),$$

where *A*_0_ is the amplitude, *f*_R_ is the beat frequency and $\varphi_{0}$ is the initial phase of the beat frequency signal. For triangular wave modulation, the ranging formula for FMCW LiDAR is given by:

(2) $$L=\frac{\tau v}{2}=\frac{vf_{R}}{2\gamma }=\frac{cf_{R}T}{4nB},$$

where $\tau =f_{R}/\gamma$ is the time delay between the target signal, the local signal γ=2B/T is the frequency sweep rate, c is the speed of light in vacuum, v=c/n is the speed of light in the medium and *n* is the refractive index of the medium. It is evident that the distance depends exclusively on the beat frequency. That is, once the beat frequency is determined, the target distance can be calculated accordingly.

In FMCW LiDAR systems, noise interference is diverse, including laser phase noise, intensity noise, environmental noise, and detector noise. Among these, Gaussian noise, primarily arising from shot and thermal noise in the photodetector, is the dominant interference source in the signal detection link under normal circumstances [21]. Such Gaussian noise exists independently and is unaffected by the presence or absence of the signal. Particularly in long-distance detection where target echo power is extremely low, it can completely submerge the weak beat frequency signal, thereby hindering accurate frequency extraction [8]. Therefore, the proposed HOIGL algorithm functions as a module within the system’s signal processing chain, aiming to robustly extract frequency information in Gaussian noise and thereby improve overall ranging performance.

### 2.2. Principle of HOIGL Algorithm

**Figure 2:**
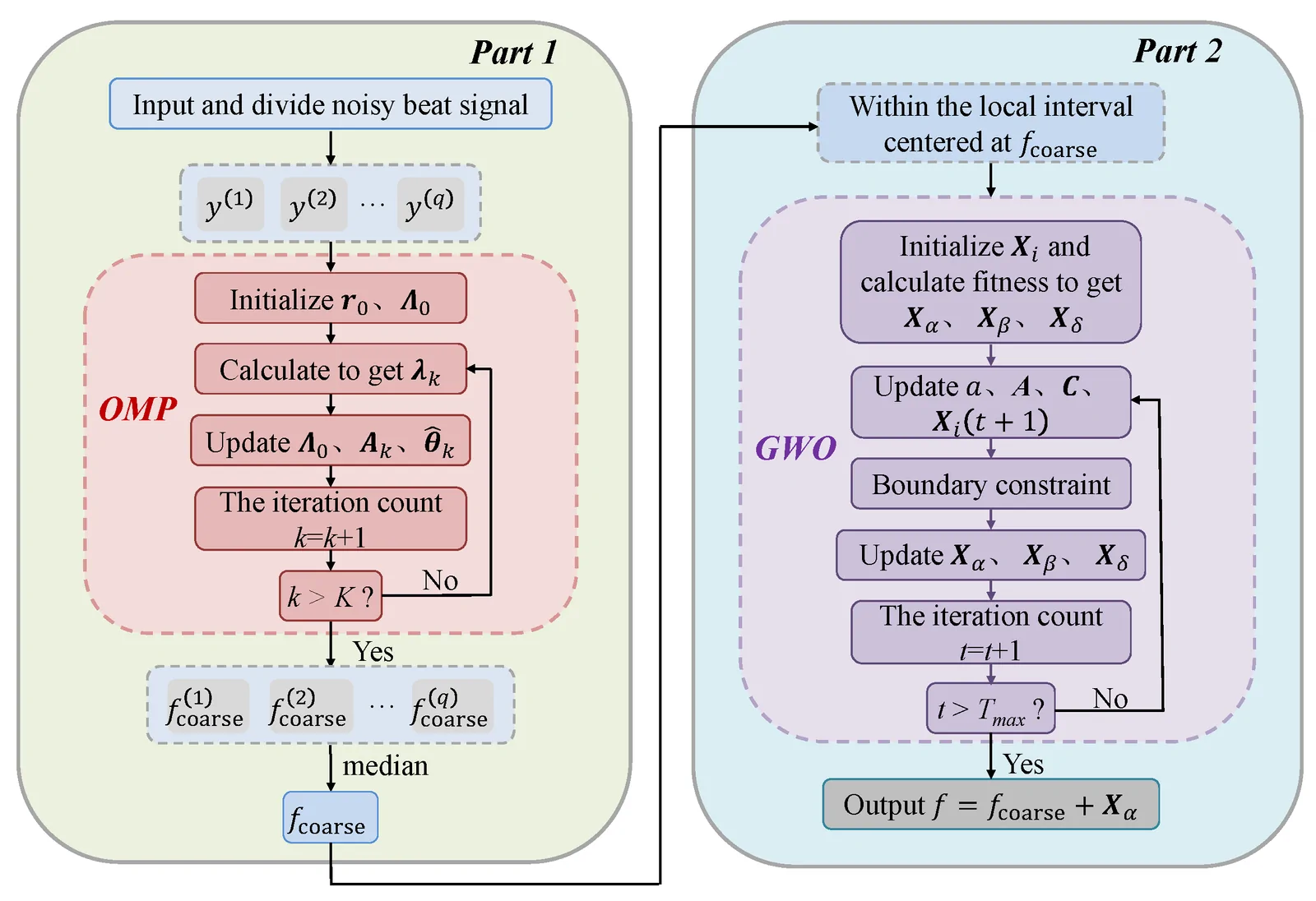
Diagram of the HOIGL algorithm.

As illustrated in Figure 2, the proposed HOIGL algorithm employs a synergistic hybrid framework that combines OMP and GWO to achieve robust frequency estimation for FMCW LiDAR under low-SNR conditions. The overall processing flow of the proposed algorithm is detailed below.

#### 2.2.1. OMP

As shown in Equation (1), when an FMCW LiDAR system measures the distance to a single target, the beat signal output by the photodetector manifests as a single-frequency sinusoidal wave. It indicates that the beat signal possesses an inherent sparsity in the frequency domain. Conversely, the Gaussian noise exhibits a broadband random distribution across the frequency domain. Leveraging this characteristic, we employ the OMP algorithm to address the aforementioned challenge. The OMP algorithm is a typical greedy iterative algorithm [19]. In each iteration, the algorithm selects the atom from the dictionary that exhibits the highest inner product with the current residual, performs orthogonal projection via the least squares method and subsequently updates the residual [22]. This process continues until a predefined sparsity or residual threshold is reached, yielding a sparse representation of the signal. Among them, the index of the selected atom corresponds to the signal frequency. Therefore, the OMP algorithm can simultaneously perform signal denoising and frequency extraction.

As illustrated in Part 1 of Figure 2, to reduce computational complexity, enhance noise resistance and suppress abnormal estimation values, we divide the beat signal into $y^{\left(q\right)}\left(q=1,2,\ldots ,Q\right)$. The OMP algorithm is then applied to each segment to perform sparse representation and obtain individual frequency estimates, $f_{\mathrm{coarse}}^{\left(q\right)}\left(q=1,2,\ldots ,Q\right)$. By calculating the median of these estimates, we derive *f*_coarse_ as a coarse initial estimate of the beat signal.

The process of OMP is as follows. For example, for the sub signal **y**^(*q*)^:

1. Input *K*, $D^{\left(q\right)}$, and $y^{\left(q\right)}$. $D^{\left(q\right)}$ is a frequency dictionary, whose j column is given by:

(3) $$\varphi_{j}^{\left(q\right)}=\cos\left(2\pi f_{j}t^{\left(q\right)}\right),f_{j}\in \left[0,f_{\max}\right],j=1,\ldots ,M$$

2. Initialize the residual $r_{0}$ and the support set $\Lambda_{0}$ as shown in Equation (4) and Equation (5).

(4) $$r_{0}=y^{\left(q\right)}$$

(5) $$\Lambda_{0}=\emptyset$$

Set the iteration count *k* = 1.

3. Calculate the absolute inner products between the current residual $r_{k-1}$ and each column vector in the $D^{\left(q\right)}$. Then, identify the index $\lambda_{k}$ corresponding to the maximum correlation value as shown in Equation (6):

(6) $$\lambda_{k}=\arg\underset{j=1,2,\ldots ,M}{\max}\left|\langle r_{k-1},\varphi_{j}^{\left(q\right)}\rangle \right|,$$

where $\varphi_{j}^{\left(q\right)}$ represents the j column of the dictionary.

4. Update the support set by adding the corresponding index $\lambda_{k}$, and construct the set of selected reconstruction atoms from the dictionary matrix, as shown in Equation (7) and Equation (8).

(7) $$\Lambda_{k}=\Lambda_{k-1}\cup \left\{\lambda_{k}\right\}$$

(8) $$A_{k}=D\left(:,\;\Lambda_{k}\right)$$

5. The sparse solution can be estimated using the least squares method, which is expressed by Equation (9).

(9) $${\hat{\theta }}_{k}={\left({A_{k}}^{T}A_{k}\right)}^{-1}{A_{k}}^{T}y$$

6. Update the residual for the next iteration as shown in Equation (10).


(10) $$r_{k}=y-A_{k}{\hat{\theta }}_{k}$$

7. Update the iteration count to k=k+1, and repeat steps 3 to 7 until the stopping criterion k≤K is met. Finally output the reconstructed signal ${\hat{\theta }}_{k}$ and the frequency of signal.

(11) $$\begin{array}{l} f_{\mathrm{coarse}}^{\left(q\right)}=f_{\lambda_{k}},\\ f_{\mathrm{coarse}}=\mathrm{median}\left({\left\{f_{\mathrm{coarse}}^{\left(q\right)}\right\}}_{q=1}^{Q}\right) \end{array}$$

**Figure 3:**
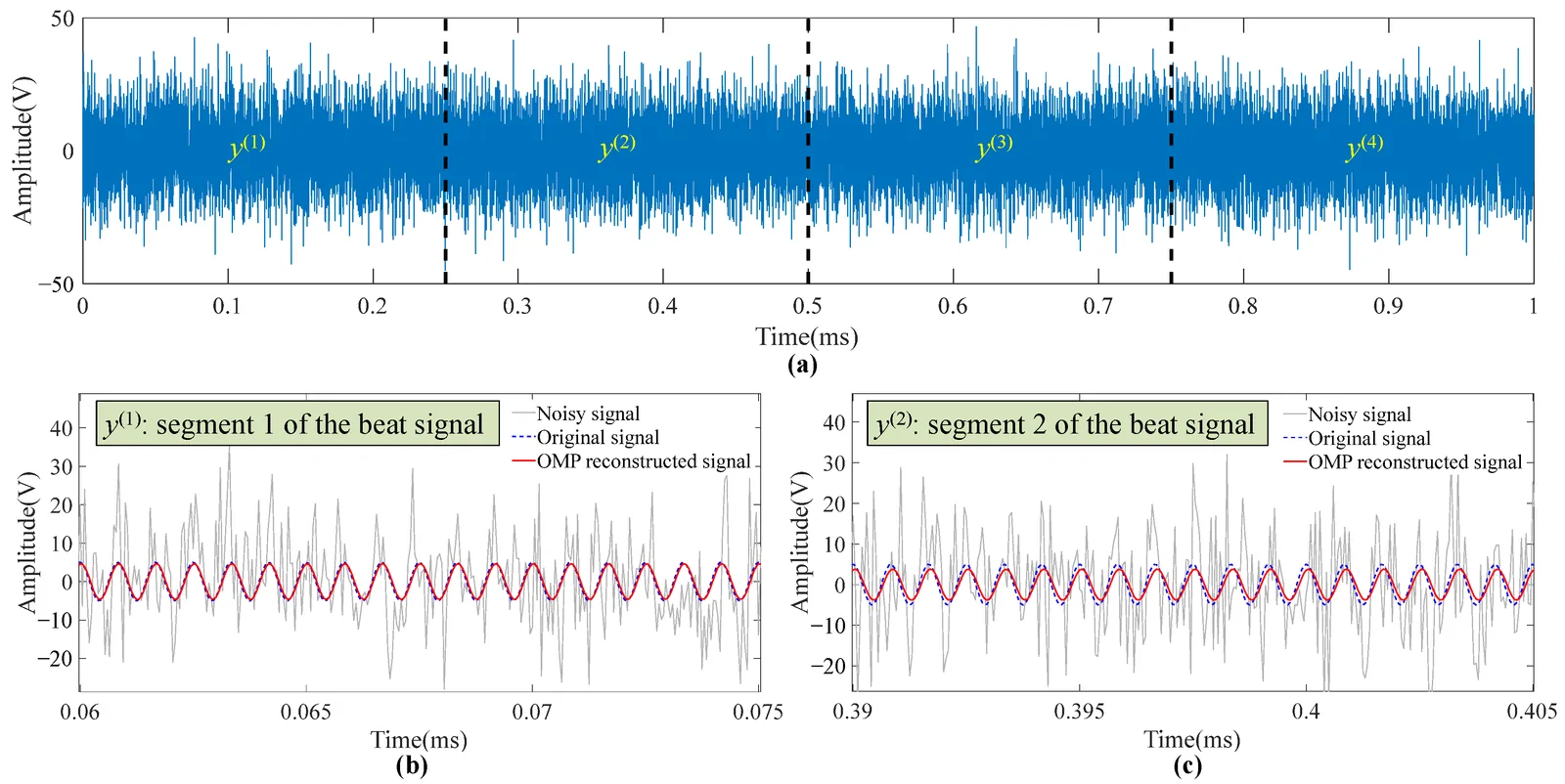

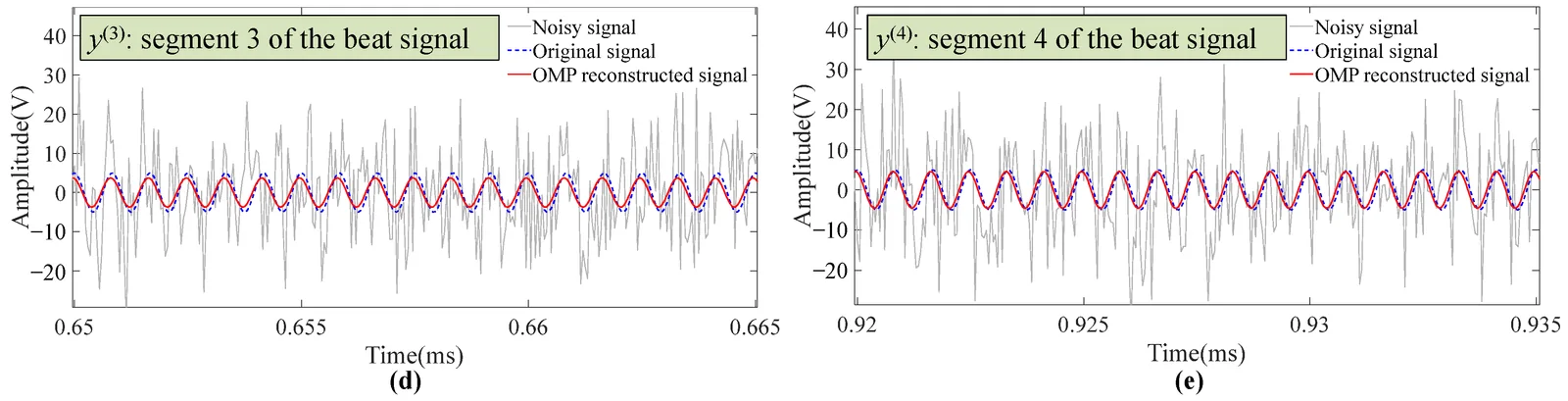
Piecewise processing of the beat signal using the OMP algorithm. (**a**) Segmentation of the noisy beat signal; (**b**–**e**) Time-domain comparison of the noisy, original, and OMP-reconstructed signals for each segment.

As illustrated in Figure 3a, the noisy beat signal is partitioned into four distinct segments, $y^{\left(1\right)}$, $y^{\left(2\right)}$, $y^{\left(3\right)}$ and $y^{\left(4\right)}$. The OMP processing effectively extracts the underlying sinusoidal components from the heavy Gaussian noise background, as evidenced by the high fidelity of the reconstructed red waveforms relative to the original blue signals across all segments in Figure 3b–e.

#### 2.2.2. GWO

However, the inherent discretization of the dictionary in the OMP algorithm inevitably introduces estimation bias. To mitigate this limitation, we integrate the GWO algorithm to refine the frequency estimation. GWO is a meta-heuristic optimization algorithm inspired by the predatory behavior of gray wolf packs [20]. The algorithm mimics the strict hierarchical system (α, β, δ, and ω) of the gray wolf pack and the collective hunting mechanisms, including surrounding, chasing, and attacking prey [23]. During the iterative process, the three leading wolves (α, β and δ) guide the rest of the pack in updating their positions. By gradually narrowing the encirclement, it achieves a balance between local search and global exploration, ultimately approaching the global optimal solution. However, an excessively large search range can lead to significant computational complexity. Therefore, GWO performs a refined continuous local search within the neighborhood interval centered at the initial frequency estimated by OMP.

In Part 2 of Figure 2, the GWO algorithm is employed to perform a refined search within the local interval centered at $f_{\mathrm{coarse}}$. Within the search interval, we assume the optimization problem to be $f=f_{\mathrm{coarse}}+X$, where X represents the frequency offset. The gray wolf population size is set as N, the current iteration count as t, and the maximum number of iterations as $\;T_{\max}$.

The processing steps of the GWO algorithm are as follows:

1. Randomly initialize candidate solutions: $\left\{\left.X_{i}\right|i=1,2,\ldots ,N\right\}$. To enhance estimation robustness, we design a composite fitness function that integrates the time-domain correlation of the signal, residual energy, and spectral peak intensity. Each candidate solution is evaluated using this function, and the gray wolf population is ranked accordingly. The top three solutions are identified as $X_{\alpha }$, $X_{\beta }$, and $X_{\delta }$, respectively. The fitness function is formulated as follows:

(12) $$\mathrm{Fitness}\left(f\right)=w_{1}\cdot \rho \left(f\right)+w_{2}\cdot \frac{1}{1+\frac{E_{\mathrm{res}}\left(f\right)}{1000}}+w_{3}\cdot P_{\mathrm{spec}}\left(f\right),$$

where $w_{1}$, $w_{2}$, and $w_{3}$ are weighting coefficients, $\rho \left(f\right)$ is the normalized correlation coefficient, $E_{\mathrm{res}}\left(f\right)$ is the residual energy, and $P_{\mathrm{spec}}\left(f\right)$ is the local spectral confidence, which are defined as shown in Equation (13).

(13) $$\left\{\begin{array}{l} \rho \left(f\right)=\left|\frac{\sum_{n=1}^{N}y_{n}\cos\left(2\pi ft_{n}\right)}{\sqrt{\sum_{n=1}^{N}y_{n}^{2}\sqrt{\sum_{n=1}^{N}\cos^{2}\left(2\pi ft_{n}\right)}}}\right|\\ E_{\mathrm{res}}\left(f\right)=\overset{N}{\underset{n=1}{\sum }}{\left(y_{n}-\cos\left(2\pi ft_{n}\right)\right)}^{2}\\ P_{\mathrm{spec}}\left(f\right)=\min\left(1,\frac{\max_{\left|f^{\prime }-f\right|<\varepsilon f}Y\left(f^{\prime }\right)}{10\cdot \mathrm{mean}\left(Y\right)}\right) \end{array}\right.,$$

where $Y\left(f^{\prime }\right)$ is the short-time FFT magnitude spectrum of the signal, ε is a scaling factor that controls the width of the local search window, $f^{\prime }$ is the frequency variable within the local neighborhood, and $\mathrm{mean}\left(Y\right)$ is the average magnitude of the entire spectrum.

2. Update the convergence factor a, the coefficient vectors A and C, respectively.

(14) $$a\left(t\right)=2-\frac{2t}{T_{\max}}$$

(15) $$A_{i}\left(t\right)=2a\left(t\right)\cdot r_{1}-a\left(t\right)$$

(16) $$C_{i}\left(t\right)=2\cdot r_{2}$$

In the above expressions, $r_{1}$ and $r_{2}$ are random vectors generated within the interval of [0,1]. The parameter $a\left(t\right)$ decreases linearly from 2 to 0 during the iteration process. The command vector, $A_{i}\left(t\right)$, can direct ω away from the prey, thereby expanding the exploration range to avoid local optima, or converge toward the prey to complete the attack behavior. $C_{i}\left(t\right)$ acts as the random perturbation, facilitating a balance between global exploration and local exploitation.

3. Update $X_{i}\left(t+1\right)$, the position of each individual.

(17) $$\left\{\begin{array}{c} D_{\alpha }\left(t\right)=\left|C_{\alpha }\left(t\right)\cdot X_{\alpha }\left(t\right)-X_{i}\left(t\right)\right|\\ D_{\beta }\left(t\right)=\left|C_{\beta }\left(t\right)\cdot X_{\beta }\left(t\right)-X_{i}\left(t\right)\right|\\ D_{\delta }\left(t\right)=\left|C_{\delta }\left(t\right)\cdot X_{\delta }\left(t\right)-X_{i}\left(t\right)\right| \end{array}\right.,$$

where $D_{\alpha }\left(t\right)$, $D_{\beta }\left(t\right)$ and $D_{\delta }\left(t\right)$ represent the distances from i to α, β and δ respectively. $C_{\alpha }\left(t\right)$, $C_{\beta }\left(t\right)$ and $C_{\delta }\left(t\right)$ denote random perturbations for α, β and δ within the interval of [0,1], while $X_{\alpha }\left(t\right)$, $X_{\beta }\left(t\right)$, $X_{\delta }\left(t\right)$ and $X_{i}\left(t\right)$ indicate the current positions of α, β and δ.

(18) $$\left\{\begin{array}{c} X_{1}=X_{\alpha }\left(t\right)-A_{1}\left(t\right)\cdot D_{\alpha }\left(t\right)\\ X_{2}=X_{\beta }\left(t\right)-A_{2}\left(t\right)\cdot D_{\beta }\left(t\right)\\ X_{3}=X_{\delta }\left(t\right)-A_{3}\left(t\right)\cdot D_{\delta }\left(t\right) \end{array}\right.$$

(19) $$X_{i}\left(t+1\right)=\frac{X_{1}+X_{2}+X_{3}}{3}$$

$X_{1}$, $X_{2}$ and $X_{3}$ represent the reference positions that i needs to adjust based on the guidance of the α, β and δ, respectively, while $X_{i}\left(t+1\right)$ denotes the final updated position of i. When the position of a gray wolf individual exceeds the search range, a dynamic boundary constraint strategy is applied as shown in Equation (20).

(20) $${X^{\mathrm{new}}}_{i}=\mathrm{sign}\left(X_{i}\right)\cdot 0.8\Delta f$$

This approach preserves the original search direction while dynamically constraining the individuals within the boundaries through proportional scaling. The boundary scaling factor is empirically set to 0.8 based on preliminary convergence testing, which provides an optimal trade-off where a higher factor (e.g., close to 1) risks frequent boundary collisions and numerical oscillations, while a lower factor excessively restricts the search space, thus ensuring a safe inner margin while maintaining sufficient local search capability. This mechanism facilitates a refined search near the predefined search limits, thereby enhancing the ability to locate the global optimum near the boundaries.

4. Update the fitness values for all individuals and update $X_{\alpha }$, $X_{\beta }$ and $X_{\delta }$. Then update the iteration count t=t+1 and repeat steps 2 to 4 until $t>T_{\max}$. The optimal frequency is given by: $f=f_{\mathrm{coarse}}+X_{\alpha }$.

**Figure 4:**
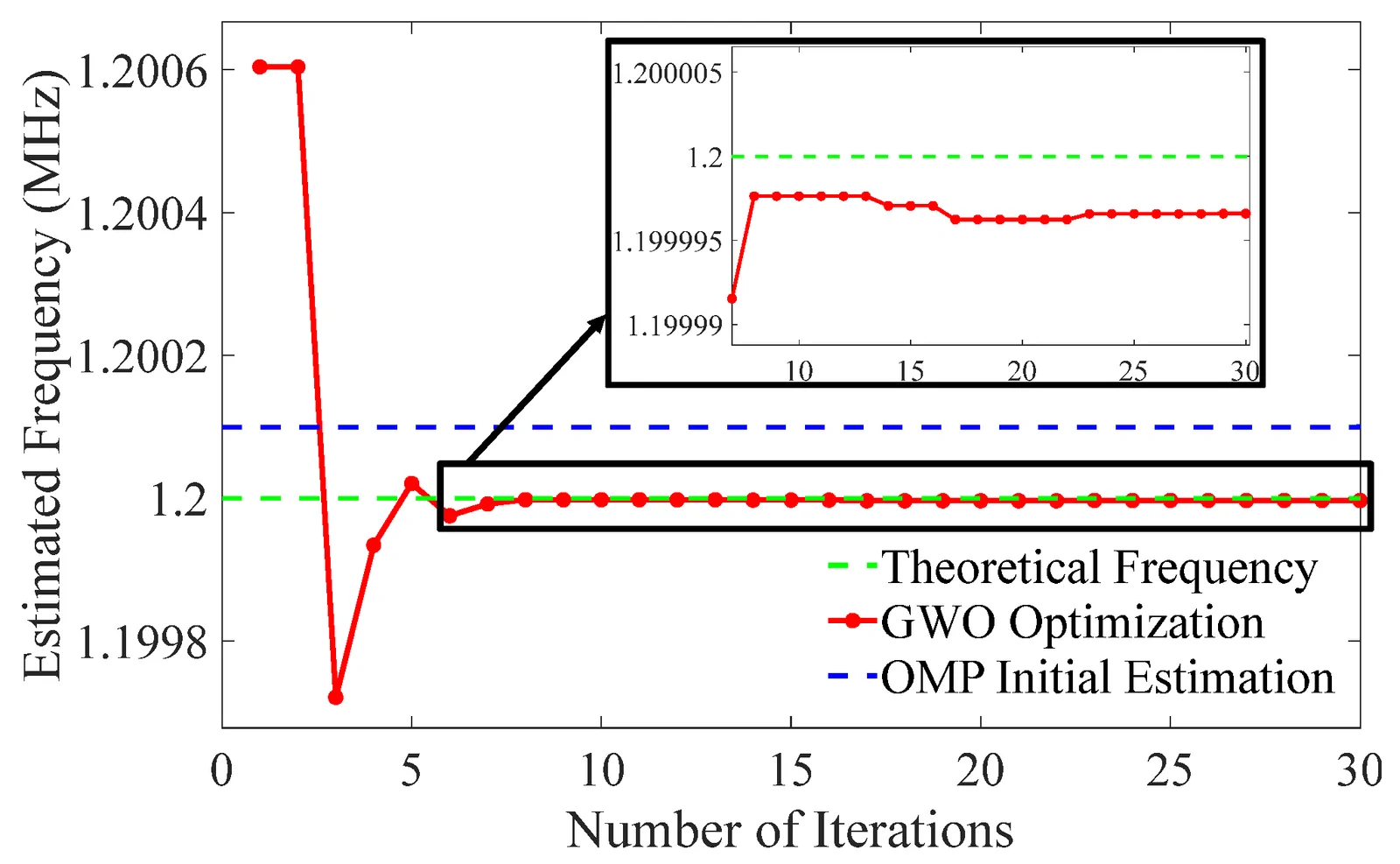
Convergence curve of the GWO optimization process compared with the theoretical frequency and OMP initial estimation under an SNR of −20 dB for a 300 m range. The global convergence process (1–30 iterations) shows that starting from the OMP initial estimate, GWO rapidly overcomes the discretization bias and converges toward the theoretical benchmark, while the zoomed-in view of the steady-state residual error in the later convergence stage (7–30 iterations) illustrates search stability and a narrow residual error bound.

To further demonstrate the optimization dynamics of the GWO algorithm under an SNR of −20 dB for a 300 m range, Figure 4 illustrates the convergence curve of the estimated frequency over iterations. As shown in Figure 4, the GWO search initializes near the OMP coarse estimate and rapidly approaches and converges toward the theoretical value within a few iterations. To examine the steady-state residual error, Figure 4 provides a zoomed-in view from iterations 7 to 30. This detailed perspective shows that the optimization maintains high search stability, rapidly stabilizing within approximately 10 iterations, and confines the residual frequency difference to a remarkably narrow bound, demonstrating the search efficiency and stability of the GWO algorithm.

**Figure 5:**
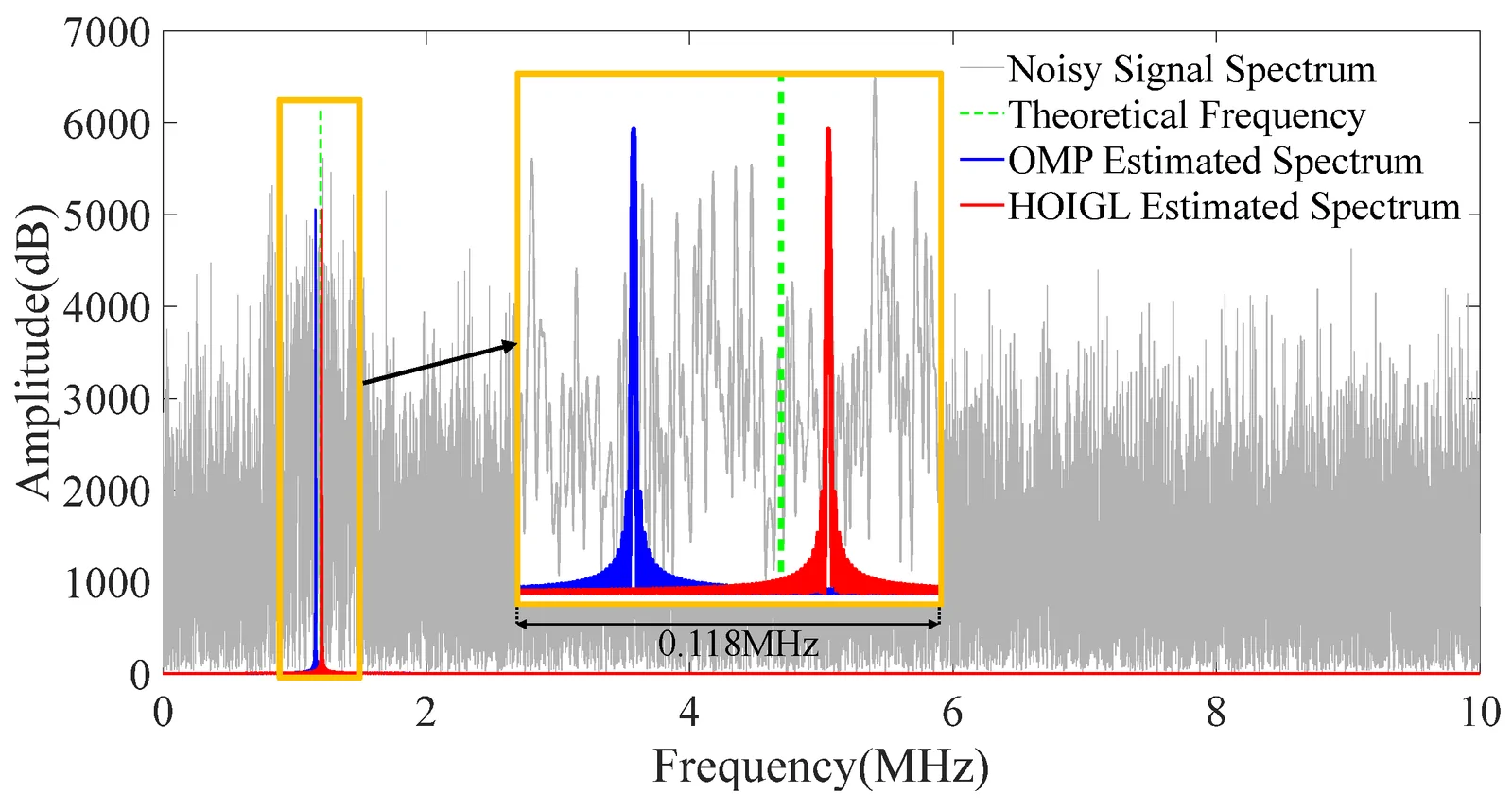
Comparison of frequency spectrum with a sampling frequency of 20 MHz and a total sample size of 20,001: the noisy signal spectrum, the theoretical frequency corresponding to 1200 cycles, the OMP coarse estimate yielding a fractional number of 1157.08 cycles, and the HOIGL final estimate of fractional 1204.22 cycles within a zoomed-in yellow local neighborhood of a 0.118 megahertz width.

Finally, Figure 5 illustrates the effectiveness of the proposed HOIGL algorithm in the frequency domain, with a sampling frequency of 20 MHz and a total sample size of 20,001 over the observation interval. As shown, the OMP stage yields an initial coarse frequency estimate corresponding to 1157.08 cycles within the time interval. To overcome the limitations of dictionary discretization, the HOIGL algorithm employs GWO to perform a refined local search. The zoomed-in yellow inset details this local neighborhood with a width of 0.118 MHz. It shows how the optimization successfully refines the fractional number of cycles to 1204.22, aligning closely with the theoretical benchmark of 1200 cycles.

## 3. Simulation Setup and Results

To evaluate the core performance and noise-suppression limits of the proposed HOIGL framework, we establish a single-target stationary scenario as the primary evaluation benchmark.

### 3.1. Simulation Setup

The simulation tests were conducted on a Windows operating system with an Intel^®^ Core™ i7-4790 CPU, an NVIDIA GT 730 GPU, and 16.0 GB of RAM. The software used was MATLAB R2019. To comprehensively evaluate the ranging performance and computational efficiency of the proposed algorithm under extreme long-range and harsh noise conditions, 200 Monte Carlo trials are conducted at the maximum detection distance of 300 m, with the SNR varying from −20 dB to 5 dB at an interval of 5 dB.

We designed the comparative experiments to analyze the root mean square error (RMSE) and ranging time of these different methods. The formula for calculating the ranging RMSE is as follows:

(21) $$S_{\mathrm{RMSE}}=\sqrt{\frac{\sum_{n=1}^{M}{\left(x_{n}-x_{\mathrm{real}}\right)}^{2}}{M}},$$

where $x_{n}$ is the ranging result, $x_{\mathrm{real}}$ is the true distance, and M is the number of Monte Carlo trials. A smaller $S_{\mathrm{RMSE}}$ indicates higher ranging accuracy of the method.

We introduced the Cramér–Rao Lower Bound (CRLB) derived with an unknown initial phase to establish a theoretical performance benchmark. The CRLB for the frequency estimation of the beat signal can be expressed as:

(22) $$\mathrm{CRLB}(f)=\frac{3f_{s}^{2}}{2\pi^{2}{\mathrm{SNR}}_{\mathrm{linear}}\cdot N_{s}({N_{s}}^{2}-1)},$$

where $f_{s}$ is the system sampling frequency, $N_{s}$ denotes the total number of sampling points of the signal and ${\mathrm{SNR}}_{\mathrm{linear}}$ denotes the linear SNR. By mapping the frequency estimation to the target range, the CRLB for the distance estimation can be expressed as:

(23) $$\mathrm{CRLB}(R)={\left(\frac{cT}{4nB}\right)}^{2}\cdot \mathrm{CRLB}(f).$$

The square root of CRLB(R) is used as the theoretical benchmark in RMSE performance comparisons.

In the simulation, the key parameters of the system, the OMP parameters and the GWO parameters set in the simulation verification are shown in Table 1.

### 3.2. Parameter Justification and Sensitivity Analysis

For the parameter configuration of the OMP stage, the window size, number of segments, and the dictionary resolution are carefully balanced to ensure local stationarity, computational efficiency, and robust anti-noise performance. Specifically, regarding the window size, setting it too large (e.g., 10,000 points) causes the temporal span to be excessively long, making it vulnerable to signal fluctuations or external interference, which severely degrades local stationarity and blurs transient frequency characteristics; conversely, setting the window size too small results in insufficient sample information per segment, leading to deteriorated frequency resolution. Therefore, a moderate window size of 5000 points is chosen to maintain optimal local stationarity. The number of segments, derived by dividing the total number of sampling points by the window size, is set to 4 to partition the signal into localized observation intervals, allowing the median-based fusion across segments to effectively suppress outlier errors induced by heavy Gaussian noise (e.g., −20 dB). Finally, the dictionary resolution is set to 1000. An excessively high resolution would drastically increase computational overhead and cause high mutual coherence between closely spaced dictionary atoms, rendering the algorithm prone to false alarms under low-SNR conditions where noise components are mistakenly selected as valid atoms. A moderate resolution of 1000 strikes an optimal balance, providing sufficient grid precision for the initial coarse estimation to guide the subsequent GWO local search while avoiding excessive computational burdens and noise-induced misclassifications. The number of OMP iterations set to 1 for the single-target FMCW scenario is theoretically determined by the designed simulation model specifications and the single-frequency beat signal model.

Regarding the population size of the gray wolf optimization stage, the population size is set to 10. A moderate population size ensures an optimal balance between search diversity and computational efficiency because a larger population size would drastically increase the computational burden per iteration without offering significant accuracy gains, whereas an overly small population would lack sufficient diversity to effectively explore the local neighborhood. Furthermore, because the GWO search is initialized within a narrow local neighborhood guided by the OMP coarse estimate rather than performing blind global exploration, a compact population of 10 individuals is entirely sufficient to locate the optimal solution. The appropriateness of the maximum iteration count set to 13 is directly justified by Figure 4, where the convergence curve and its zoomed-in steady-state view under a −20 dB SNR demonstrate that the algorithm rapidly approaches the theoretical benchmark and stabilizes within approximately 10 iterations, confirming that 13 iterations provide sufficient convergence margin while avoiding redundant computational overhead. Setting the local search interval excessively wide beyond plus or minus 0.05 not only risks distracting the GWO algorithm with noise-induced false peaks and increasing ranging errors, but also significantly degrades computational efficiency, whereas an overly narrow interval restricts exploration and fails to fully correct the OMP discretization bias. Furthermore, the scaling factor of 0.02 used in the spectral peak validation to define the local frequency neighborhood achieves an essential balance, as a larger factor broadens the bandwidth excessively and inadvertently incorporates surrounding noise or side-lobes that distort the peak confidence metric, while an overly narrow factor risks excluding the true frequency component under severe low-SNR spectral broadening.

**Figure 6:**
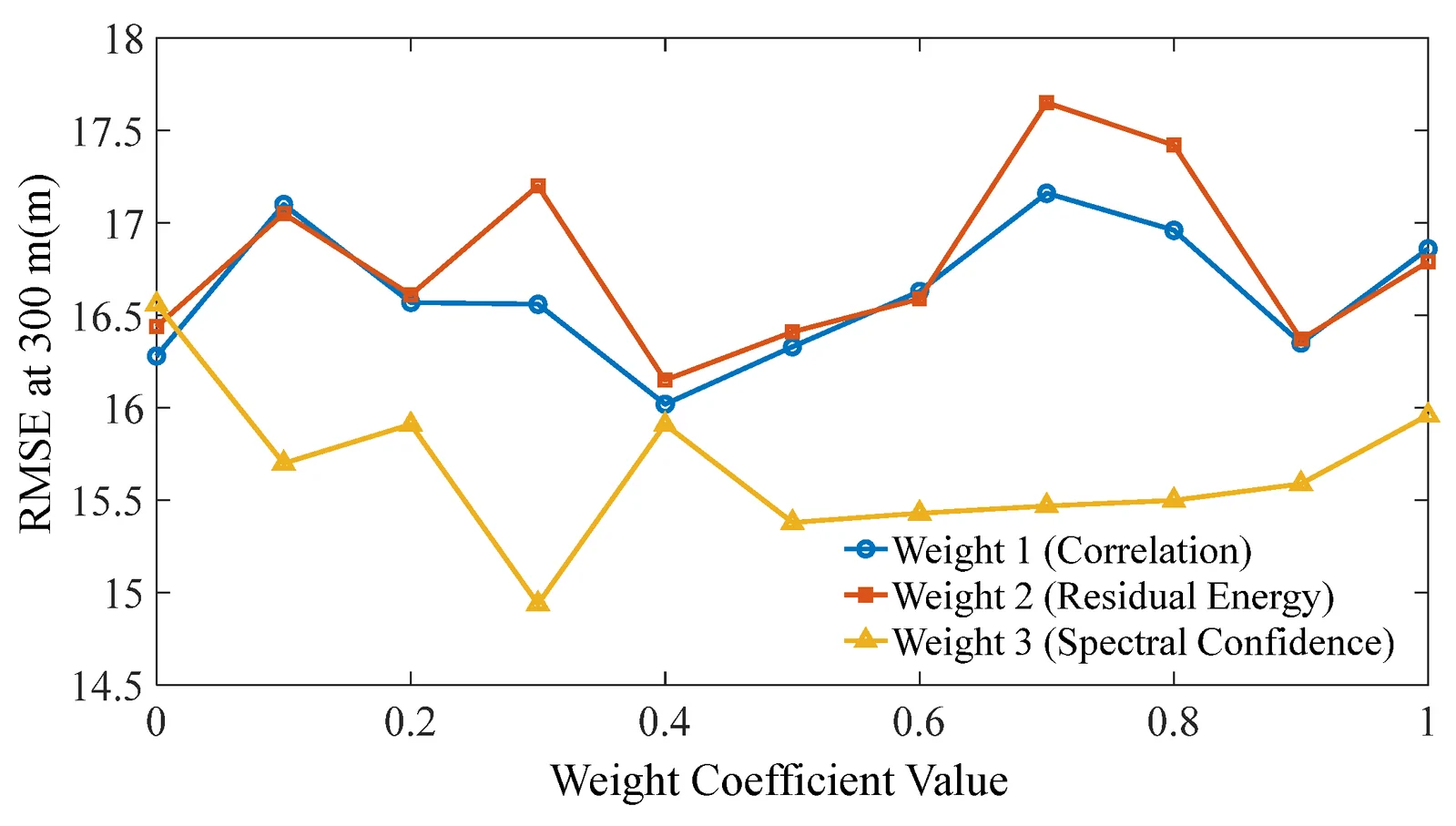
Sensitivity analysis of the fitness function weight coefficients on the ranging RMSE at a 300 m range under −20 dB noise conditions.

To analyze the impact of the weighting coefficients in the composite fitness function, each weight was independently varied from 0 to 1 to observe the resulting ranging RMSE at 300 m under low-SNR conditions, as shown in Figure 6. The performance variations for time-domain correlation $w_{1}$, residual energy $w_{2}$, and spectral peak confidence $w_{3}$ are illustrated in the sensitivity curves. The results indicate that error fluctuations occur across different weight proportions, reflecting the algorithm’s response to changes in the composite metric formulation. Based on the error minimums and overall trends observed for each curve during the evaluation, the final weight coefficients are set to 0.4 for correlation, 0.4 for residual energy, and 0.2 for spectral confidence.

**Figure 7:**
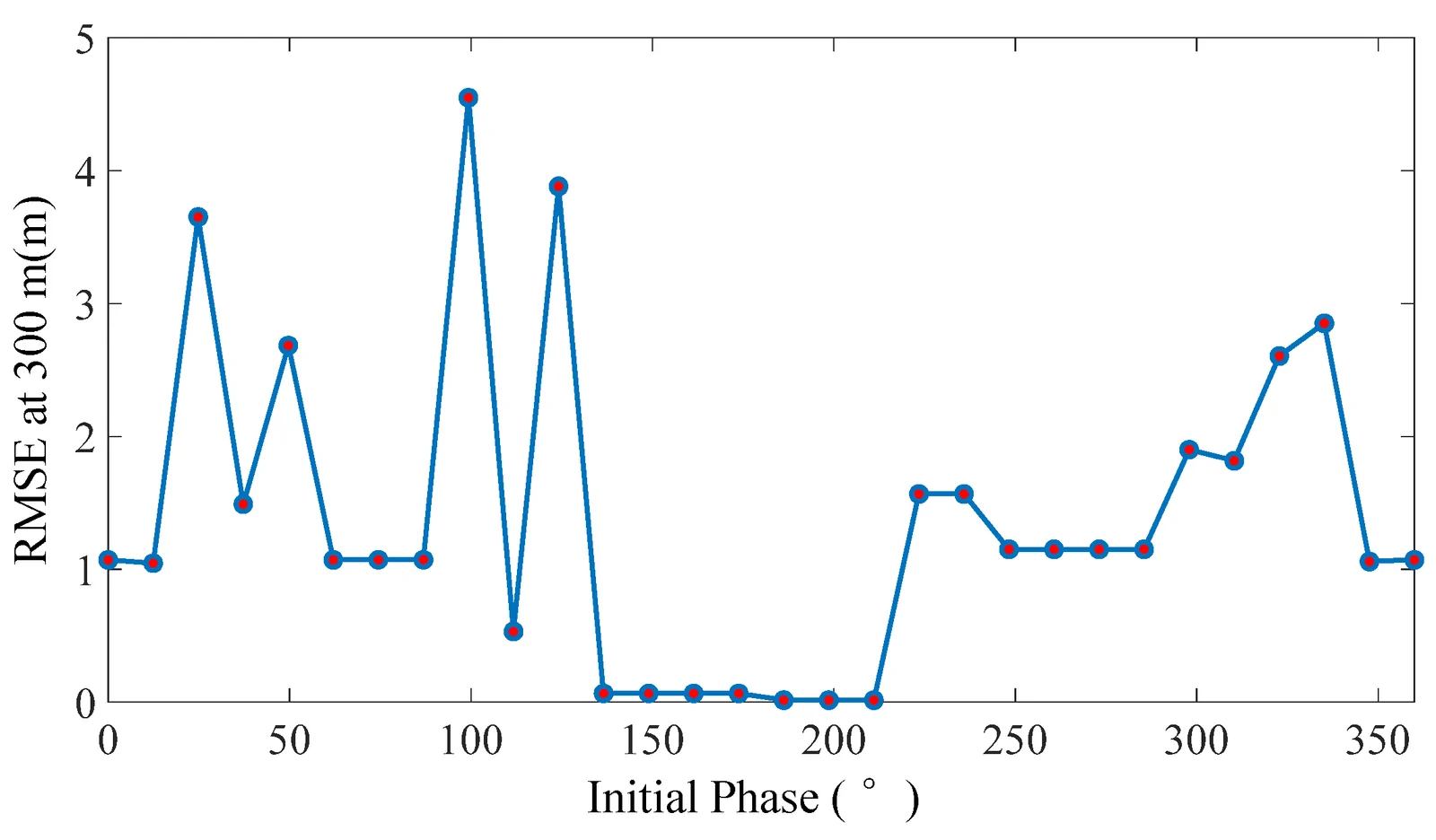
RMSE performance of the HOIGL algorithm with varying initial phase values evaluated at a 300 m distance under −20 dB noise conditions.

Considering that the unknown initial phase in Equation (1) and the fixed-phase cosine atoms of the OMP dictionary in Equation (3) could theoretically cause the inner product to vary substantially and thereby affect both coarse frequency selection and subsequent optimization, we provide a detailed analysis of how the proposed framework ensures robustness against arbitrary initial phases. In the OMP coarse estimation stage, the signal is partitioned into multiple segments and the final coarse frequency is derived by taking the statistical median of the segment-wise estimates. While localized phase mismatches may induce minor variations in individual inner products, the median operation effectively filters out such outliers and stabilizes initial guidance. Within the GWO refinement stage, although the time-domain correlation and residual energy terms in the composite fitness function are sensitive to phase shifts, they are coupled with phase-insensitive frequency-domain magnitude characteristics. This complementary design balances phase-induced fluctuations and prevents the optimization process from being derailed. Finally, to empirically validate this robustness across a broad phase range, a dedicated Monte Carlo simulation was conducted at a 300 m distance under an extreme −20 dB SNR condition by uniformly sweeping the initial phase from 0 to 2π. As illustrated in Figure 7, despite some fluctuations caused by severe noise, the maximum RMSE remains within an acceptable range, confirming the algorithm’s overall stability against arbitrary initial phases.

### 3.3. Performance Evaluation and Analysis

Building upon this simulation setup, to comprehensively evaluate the performance of the proposed HOIGL algorithm, we compare it to several classical denoising and frequency estimation algorithms under extreme long-range conditions, and further conduct an ablation study to validate the effectiveness of its core components.

#### 3.3.1. Comparison with Denoising Algorithms

**Figure 8:**
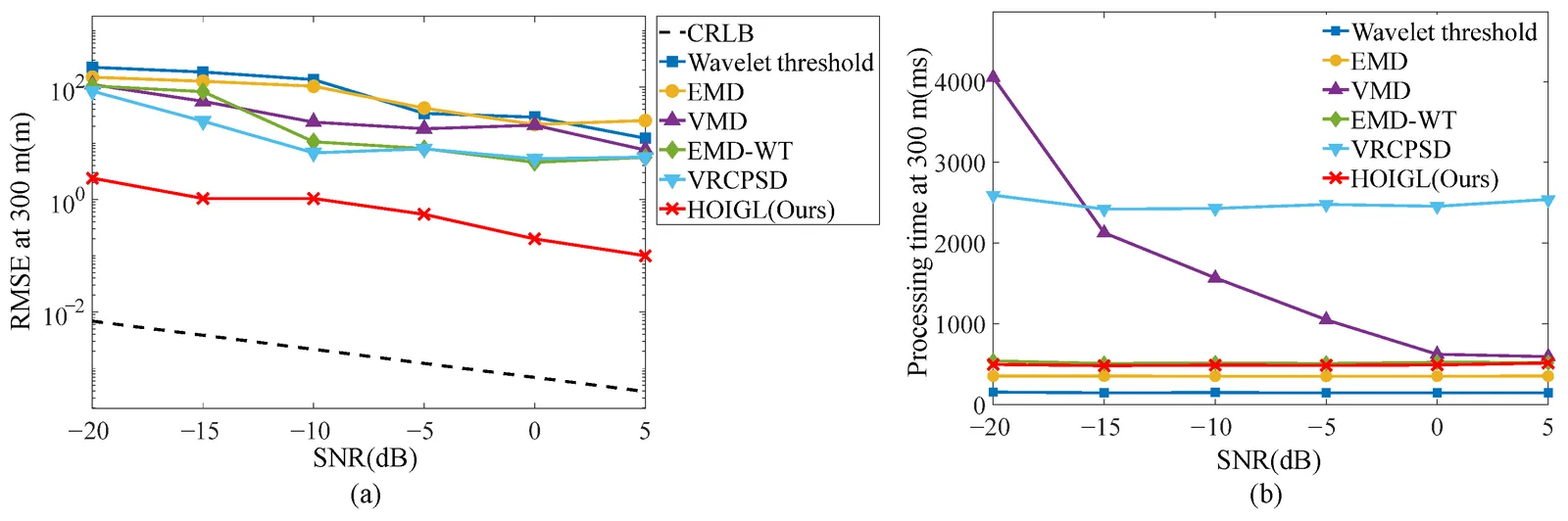
Comparison of the comprehensive performance of different denoising algorithms for measuring a 300 m distance under various SNRs. (**a**) RMSE comparison of distance measurement along with the CRLB; (**b**) Comparison of processing times.

We compare the proposed HOIGL algorithm to wavelet threshold denoising, EMD, and VMD algorithms, as well as recently proposed improved joint denoising algorithms from existing literature like EMD-WT and VRCPSD. For some algorithms requiring spectrum analysis, the FFT algorithm is uniformly applied. The simulation results comparing the RMSE and computational efficiency of different algorithms are shown in Figure 8. Under a unified timing protocol implemented via MATLAB’s tic and toc functions, the execution time for all competing algorithms was measured by capturing the entire processing cycle of each trial from signal input to final frequency estimation.

As observed in Figure 8a, the RMSE of all algorithms decreases as the SNR increases, following a downward trend above the CRLB. Classical algorithms including the wavelet threshold, EMD, and VMD methods exhibit relatively large errors, especially under low-SNR conditions. In contrast, advanced joint denoising algorithms from existing literature like EMD-WT and VRCPSD as well as the proposed HOIGL algorithm achieve significantly higher ranging accuracy. Notably, the HOIGL clearly outperforms EMD-WT and VRCPSD across all tested SNR levels, demonstrating superior anti-noise capability with a remarkably lower and stable RMSE. While all denoising algorithms suffer from an early threshold breakdown, the proposed HOIGL algorithm defers the threshold breakdown point to a much lower SNR, maintains a steadily decreasing error trend well below competing methods. Specifically, at the most challenging SNR of −20 dB, the proposed HOIGL algorithm achieves an RMSE of 2.63 m, which is significantly lower than 103.8 m of EMD-WT and 84.34 m of VRCPSD. Furthermore, when the SNR increases to 5 dB, the HOIGL algorithm achieves a very low error of only 0.1001 m, whereas the best performance among the other competing algorithms still exceeds 5.5 m.

As shown in Figure 8b, when evaluated under the same conditions, VRCPSD and VMD suffer from excessively high processing times, reaching up to 2.593 s for VRCPSD. In contrast, the HOIGL algorithm maintains a very low and stable processing time of around 0.498 s, thus achieving an exceptional balance between high ranging accuracy and high computational efficiency. Regarding computational complexity, the overall processing overhead is primarily driven by two main stages, which are the initial sparse representation stage and the subsequent local optimization search stage. The first OMP stage involves matching the signal against a structured dictionary across multiple segments, where the computational load scales with the data length and dictionary size. The subsequent GWO stage relies on an iterative search process, which is strictly controlled by employing a small population and a limited number of iterations, thereby effectively bounding the heavy search overhead typically associated with metaheuristic algorithms.

#### 3.3.2. Comparison with Frequency Estimation Algorithms

**Figure 9:**
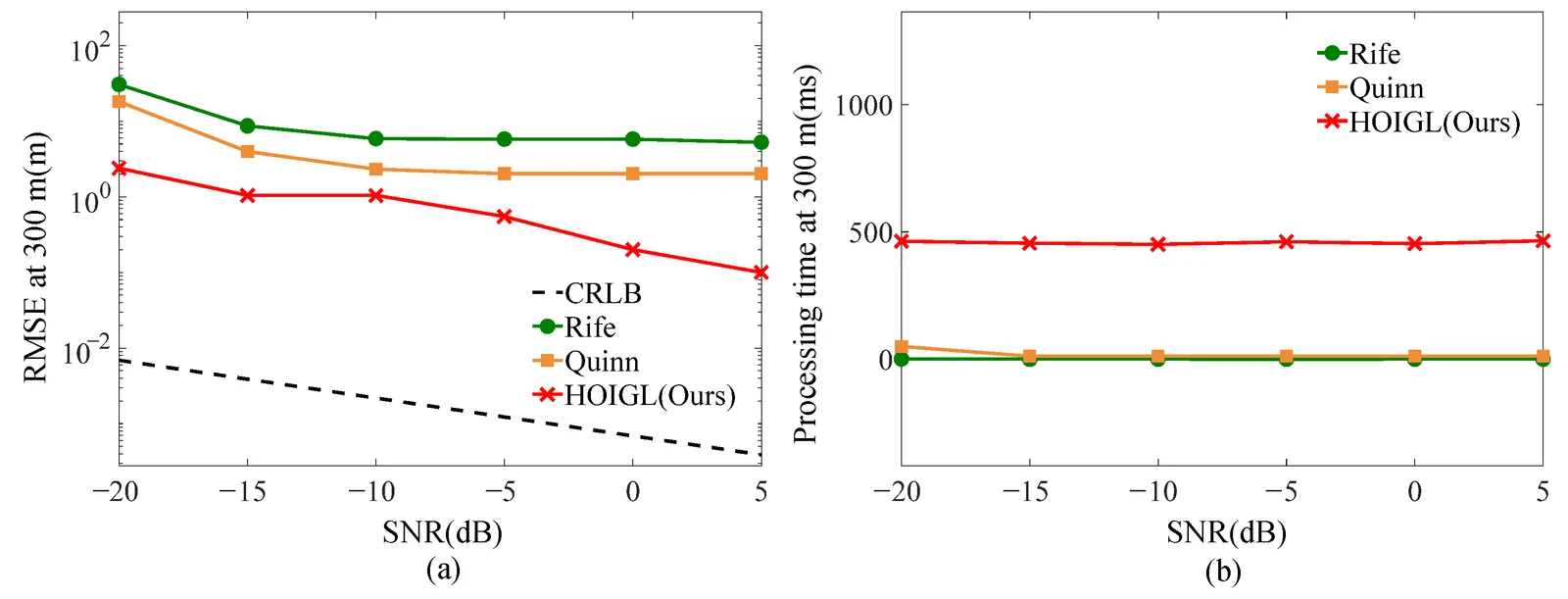
Comparison of the comprehensive performance of different frequency-estimation algorithms for measuring a 300 m distance under various SNRs. (**a**) RMSE comparison of distance measurement along with the CRLB; (**b**) Comparison of processing times.

We compare the proposed algorithm to classic frequency-estimation baselines, including the Rife algorithm and the Quinn algorithm. The simulation results comparing the root mean square error and computational efficiency of these different algorithms are shown in Figure 9.

As observed in Figure 9a, the RMSE of all algorithms decreases as the SNR increases. Although the Quinn algorithm outperforms the Rife algorithm, both classical methods exhibit prominent performance floors at moderate-to-high SNRs. In contrast, the HOIGL algorithm achieves a substantially lower RMSE, following a consistent downward trend above the CRLB across the tested SNR range. As shown in Figure 9b, while the Rife and Quinn algorithms exhibit very low computational overhead, the HOIGL maintains a stable execution time of roughly 498 ms across all evaluated SNR conditions. This indicates that the HOIGL algorithm secures a major enhancement in ranging accuracy while keeping the computational cost within a predictable range.

#### 3.3.3. Ablation Study on the HOIGL

**Figure 10:**
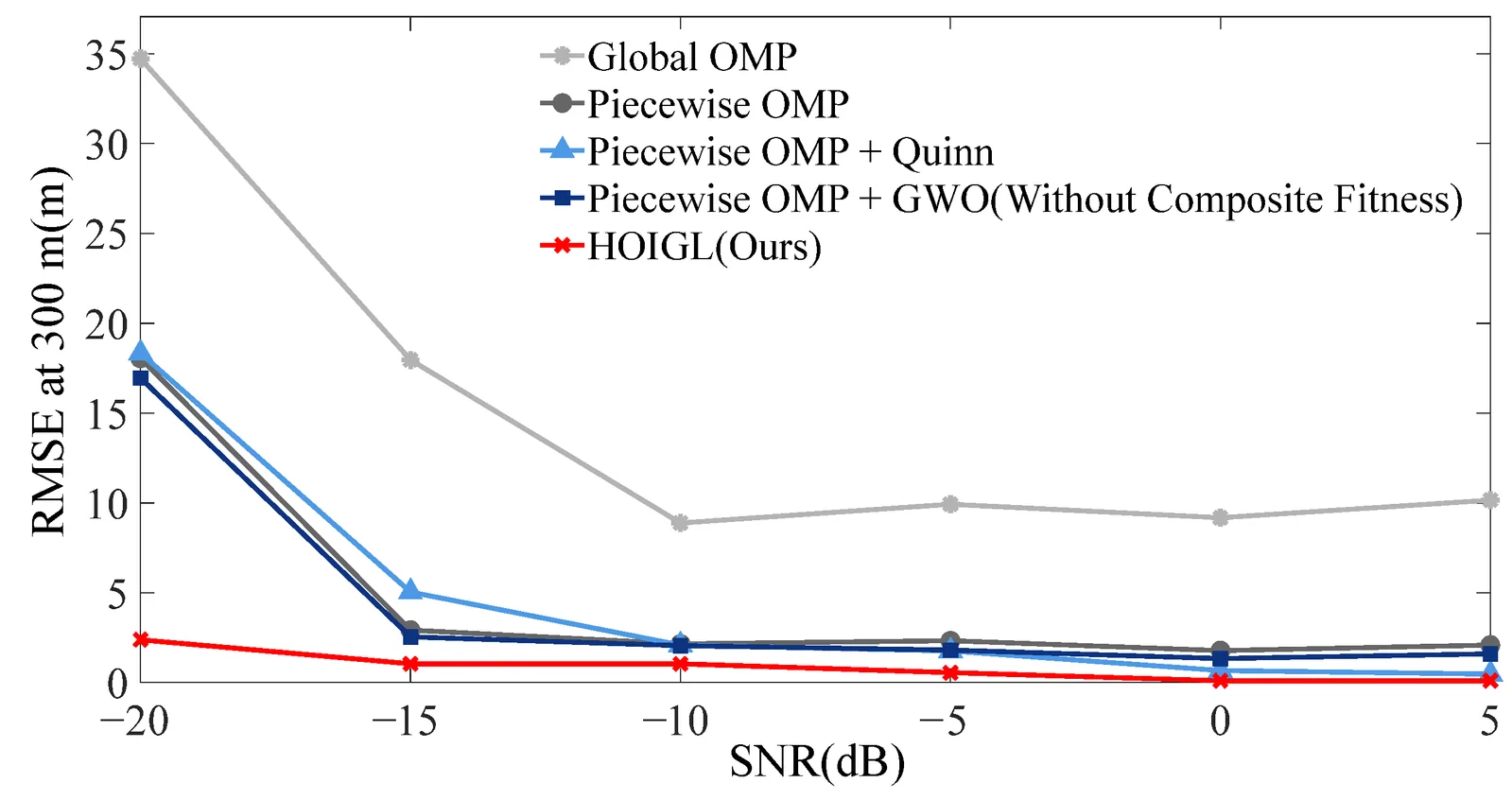
Performance comparison of ablation variants within the HOIGL framework under various SNRs.

To evaluate the contribution of each core component within the proposed HOIGL framework, we conducted a series of ablation experiments illustrated in Figure 10. The evaluation compares the performance of five distinct configurations across various SNR levels, including Global OMP, Piecewise OMP, Piecewise OMP combined with Quinn, Piecewise OMP with GWO omitting the composite fitness function, and the complete HOIGL algorithm.

As observed from the curves, the Global OMP baseline exhibits the highest RMSE across the entire SNR range, particularly suffering from severe performance degradation under low-SNR conditions such as reaching up to 34.74 m at −20 dB. Transitioning to Piecewise OMP substantially reduces the error, demonstrating the fundamental advantage of the piecewise strategy in mitigating error accumulation. Incorporating the Quinn algorithm or the standard GWO without composite fitness brings further localized improvements, yet their RMSE curves still remain relatively high under challenging noise environments. In contrast, the complete HOIGL algorithm incorporating all proposed modules including the composite fitness function consistently achieves the lowest RMSE across all tested SNR levels. Notably, HOIGL maintains exceptional stability and accuracy even at the most severe noise level of −20 dB, validating that each designed component contributes synergistically to the enhanced anti-noise robustness and precision of the overall system.

To evaluate the individual contributions of the components within the composite fitness function, we conducted an ablation analysis on the three fitness terms, namely the correlation $\rho \left(f\right)$, residual energy $E_{\mathrm{res}}\left(f\right)$, and spectral confidence $P_{\mathrm{spec}}\left(f\right)$. As summarized in Table 2, evaluating these terms individually or through the time-domain combination of correlation and residual energy fails to suppress the severe errors when measuring a 300 m distance under the challenging −20 dB noise condition, resulting in high RMSE values around 16 m. In contrast, the complete composite fitness function leverages the synergistic interaction of all three terms, drastically reducing the RMSE to 2.63 m. This demonstrates that each fitness component plays an indispensable role in ensuring robust parameter estimation under heavy noise.

## 4. Experimental Setup and Results

### 4.1. Experimental Setup

**Figure 11:**
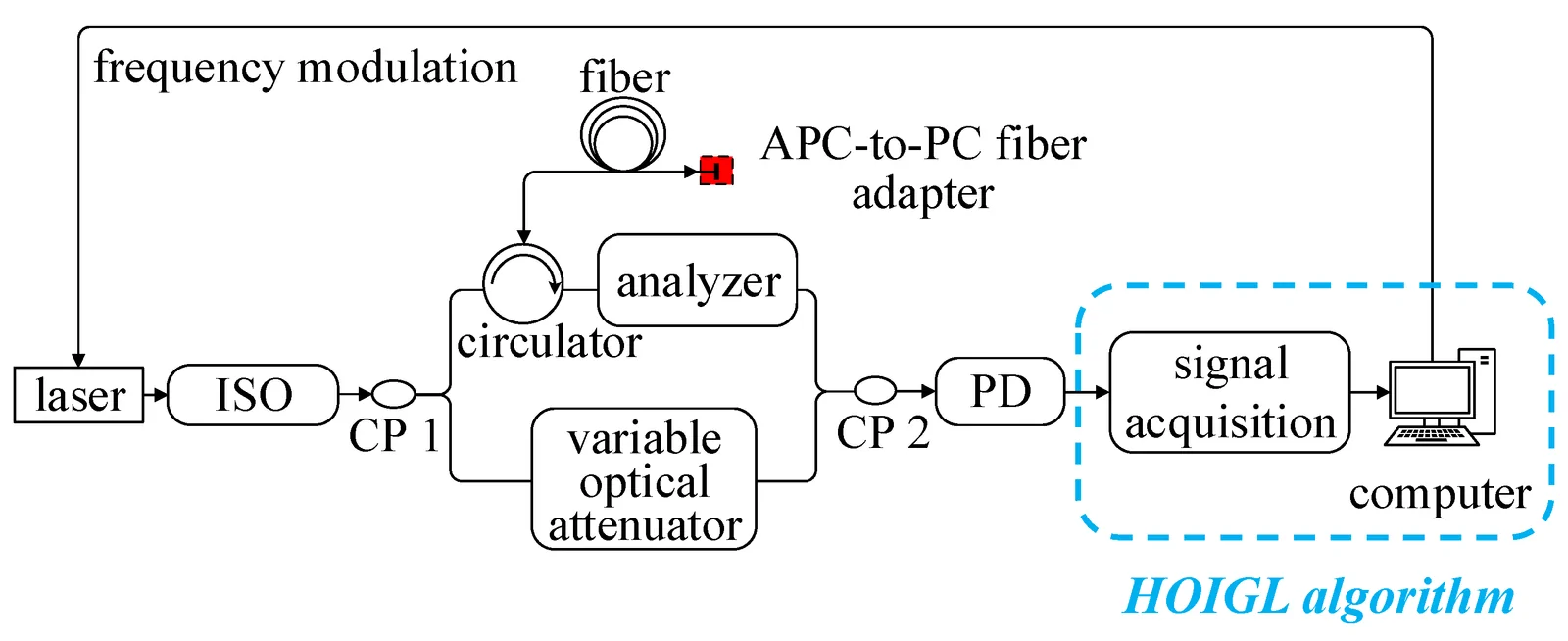
Schematic diagram of the FMCW LiDAR ranging experimental setup.

To validate the performance of the HOIGL algorithm a controlled optical-path delay, experimental measurements using various optical fiber lengths were conducted based on the setup illustrated in Figure 11. It should be noted that while minor differences exist between the experimental and theoretical simulation parameters such as period and bandwidth owing to hardware specifications, both configurations are governed by the same FMCW ranging and beat frequency mathematical model. This fiber-based configuration provides a controlled and repeatable optical path delay to simulate fixed target distances, thereby ensuring reliable and reproducible conditions for validating the ranging accuracy and signal processing algorithms. Specifically, the fiber end is terminated with an APC-to-PC fiber adapter, where the PC port is exposed to air. The resulting return loss is approximately 40 dB, thereby generating a weak target signal to serve as a controlled and repeatable optical-path-delay validation for assessing the algorithm’s signal-processing performance under low-SNR conditions.

The computer controls a distributed feedback (DFB) laser to generate linearly frequency-modulated light, which passes through an optical isolator (ISO) and is then equally split into two paths by the first 50:50 coupler (CP 1). One path enters an optical circulator and propagates through the long-distance optical fiber terminated with an APC-to-PC fiber adapter. The other path serves as the local oscillator signal and passes through a variable optical attenuator. Meanwhile, the target signal from the circulator passes through an analyzer, after which it is coherently mixed with the local oscillator signal via the second 50:50 coupler (CP 2). The resulting beat signal is converted into an electrical signal by a photodetector (PD). A data acquisition device then samples the beat signal and transmits it to the host computer for further analysis and processing. The key parameters of the main devices used in the system are listed in Table 3. In the fiber-based experiments, the ground-truth distance is determined by the physical length of the optical fiber. As defined in Equation (2), the ground-truth distance of the experiments is calculated using the triangular-wave FMCW ranging model, where the fiber’s effective group refractive index and round-trip propagation delay are explicitly incorporated.

### 4.2. Experimental Results

To evaluate performance across different spatial scales, experiments were conducted at four fiber lengths: 50 m, 100 m, 200 m, and 300 m. We quantitatively evaluated the SNR of the measured experimental beat signals.

(24) $${\mathrm{SNR}=10\log}_{10}\left(\frac{P_{\mathrm{signal}}}{P_{\mathrm{noise}}}\right),$$

where $P_{\mathrm{signal}}$ and $P_{\mathrm{noise}}$ represent the peak power of the beat signal and the average power of the background noise floor, respectively. Under the experimental configuration with a laser launch power of 18.78 dBm and a fixed 40 dB return loss, the measured experimental SNR values corresponding to the tested fiber distances of 50 m, 100 m, 200 m, and 300 m are −14.39 dB, −14.63 dB, −15.05 dB, and −15.87 dB, respectively. These values consistently reside within the severe low-SNR regime, closely aligning with the extreme low-SNR boundaries evaluated in our Monte Carlo simulations from −20 dB to 5 dB. Furthermore, varying the distance from 50 m to 300 m validates the algorithm’s robustness against both harsh noise and frequency-dependent variations across different operational ranges.

**Figure 12:**
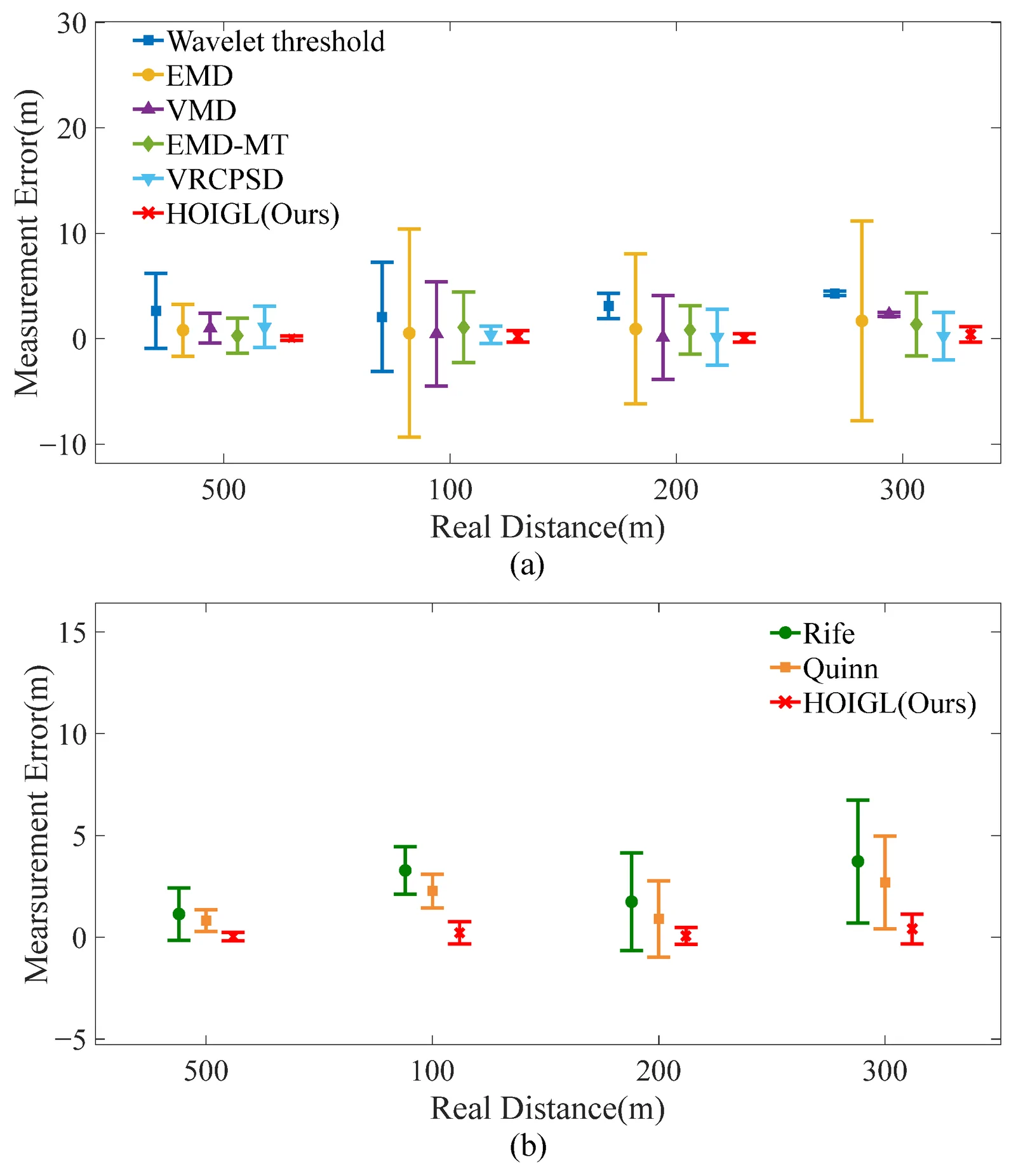
Comparison of the mean absolute error (MAE, represented by center markers) and standard deviation (SD, represented by error bars) of different algorithms in estimating various distances. (**a**) Comparison with traditional denoising algorithms; (**b**) Comparison with frequency estimation algorithms.

Each measurement was repeated ten times to improve accuracy and reduce the impact of random errors, enabling the calculation of reliable average values. As shown in Figure 12, the algorithms were comprehensively evaluated through two comparative sets of experiments, the comparison with traditional denoising algorithms in Figure 12a and the comparison with conventional frequency estimation algorithms in Figure 12b, alongside the HOIGL method proposed in this paper. Based on these repeated experiments, we calculated the mean absolute error (MAE) and its standard deviation (SD) for each algorithm. In Figure 12, the center point reflects the accuracy of the algorithm, which represents the deviation in the measured mean from the true value, while the error bars reflect the precision, indicating the dispersion of multiple measurement results.

From Figure 12, it can be seen that the HOIGL algorithm achieves the lowest MAE at each distance, and its SD remains consistently small, indicating that this algorithm has good measurement stability while ensuring high accuracy. Due to the large practical sample size of one million points, the signal processing time required to obtain the result is approximately 3.3 s per measurement. Future work will focus on algorithmic optimization and hardware acceleration to reduce execution latency.

The current validation focuses on single-target in the controlled fiber-optic link, which does not account for complex environmental factors such as atmospheric turbulence, spatial beam divergence and free-space geometric path loss. The underlying methodology combining OMP sparse representation and GWO local refinement is fundamentally generalizable to multi-component signals. Building upon this single-target benchmark, future work will extend the sparsity level and optimize the fitness function to handle complex multi-target scenarios in free-space environments.

## 5. Conclusions

In this study, the HOIGL algorithm is proposed to enhance the long-distance ranging accuracy of FMCW LiDAR under low-SNR conditions dominated by Gaussian noise. By integrating OMP’s piecewise sparse representation for robust initial value guidance with GWO’s refined continuous local search, the proposed method bypasses the traditional multi-stage processing of separate denoising and spectral analysis, thereby preventing stage-by-stage error accumulation and excessive computational complexity while delivering robust long-range ranging performance. Both simulation and experimental results thoroughly validate the feasibility and superiority of the algorithm. In MATLAB Monte Carlo simulations, compared to traditional wavelet thresholding, EMD, and VMD algorithms, as well as recent methods like VRCPSD and EMD-WT, the HOIGL algorithm achieves an RMSE of 2.63 m compared to over 84.34 m for other methods at −20 dB SNR, while maintaining a low and stable processing time of around 0.498 s. Furthermore, fiber-optic-link experimental tests across multiple ranging distances from 50 m to 300 m demonstrate that the HOIGL algorithm achieves the lowest mean absolute error and consistently small standard deviations, proving its reliable measurement stability and high accuracy under simulated low-SNR optical conditions. These findings collectively highlight the potential of the proposed method for accurate target distance measurement at hundred-meter-scale ranges. Building upon the current single-target validation in controlled fiber-optic links, future work will focus on extending the framework to handle complex multi-target scenarios, further optimizing computational efficiency, and gradually advancing the testing toward free-space atmospheric environments.

## Figures and Tables

**Table 1:** Simulation system, the OMP and the GWO parameter.

	Parameters	Values
Simulation system	number of signal samples $N_{s}$	20,001
	sampling rate $f_{s}$	20 MHz
	modulation bandwidth B	100 MHz
	modulation period T	0.5 ms
OMP	dictionary size M	1000
	window size	5000
	the number of segments Q	4
	number of OMP iterations/sparsity level K	1
GWO	population size N	10
	maximum iterations $\;T_{\max}$	13
	local search interval	$\pm 0.05f_{\mathrm{coarse}}$
	weighting coefficients $w_{1}$, $w_{2}$, $w_{3}$	0.4, 0.4, 0.2
	scaling factor ε	0.02

**Table 2:** Ablation analysis of the composite fitness function components for measuring a 300 m distance under −20 dB SNR.

	Correlation ρ(f)	Residual Energy $E_{\mathrm{res}}(f)$	Spectral Confidence $P_{\mathrm{spec}}(f)$	RMSE at −20 dB (m)
1	√			16.58
2		√		16.46
3			√	15.28
4	√	√		15.91
5	√	√	√	2.63

**Table 3:** Some of the key parameters in the system.

Parameter	Value
Laser wavelength (nm)	1550
Laser linewidth (kHz)	200
Modulation period (s)	0.004
Modulation bandwidth (MHz)	289.8
Detector response bandwidth (GHz)	10
Detector responsivity (A/W)	0.9
Refractive index of optical fiber	1.467
number of signal samples	1,000,000
Sampling Rate (MHz)	50

## Data Availability

The original contributions presented in this study are included in the article. Further inquiries can be directed to the corresponding author.
